# Psilocybin: Systematic review of its use in the treatment of depression

**DOI:** 10.1192/j.eurpsy.2025.926

**Published:** 2025-08-26

**Authors:** P. Andres-Olivera, J. de la Iglesia, E. Dominguez-Alvarez, P. Soto’Gonzalez, C. P. Rodriguez, C. Munaiz-Cossio, R. K. Gonzalez-Bolaños, R. Brito-Rey, C. Marín-Lorenzo, B. Arribas-Simon

**Affiliations:** 1 Psichiatry, CAUSA; 2 Medicine; 3 USAL; 4 CAUSA, Salamanca; 5 Psichiatry, HCUV, Valladolid, Spain

## Abstract

**Introduction:**

Psilocybin, a psychedelic compound, has shown potential in treating depression, especially in cases resistant to conventional treatments. This study systematically reviews the scientific literature to assess its efficacy and safety.

**Objectives:**

The main objective of this study is to evaluate the therapeutic effects of psilocybin for the treatment of depressive disorder through a systematic review of the current scientific literature

**Methods:**

An exhaustive search was conducted in databases such as PubMed and Web of Science, using specific MeSH term and selecting studies published between 2019-2024 that investigated the effects of psilocybin in treating depression.

**Results:**

The included studies demonstrated significant improvements in depressive symptoms with psylocibin compared to standard treatments. Studies reports a rapid and sustained symptom reduction, with few adverse effects.

**Conclusions:**

Psilocybin could be an effective and safe alternative for treating depression, providing symptomatic relief with fewer treatment sessions and a favorable safety profile. However, further research is needed to overcome current limitations and fully understand its therapeutic potential and underlying mechanisms.

**Disclosure of Interest:**

None Declared

